# Study protocol: maintaining preventive care during public health emergencies through effective coordination

**DOI:** 10.1186/s43058-023-00507-2

**Published:** 2023-11-27

**Authors:** Sylvia J. Hysong, Traber Davis Giardina, Jennifer Freytag, Richard SoRelle, Daniel R. Murphy, Jeffrey A. Cully, Yvonne H. Sada, Amber B. Amspoker

**Affiliations:** 1 Center for Innovations in Quality Effectiveness and Safety, Michael E. DeBakey VA Medical Center, Houston, TX USA; 2 Department of Medicine – Health Services Research, Baylor College of Medicine, Houston, TX USA; 3 Department of Medicine – Internal Medicine, Baylor College of Medicine, Houston, TX USA; 4 Department of Psychiatry and Behavioral Sciences, Baylor College of Medicine, Houston, TX USA; 5 Department of Medicine – Hematology/Oncology, Baylor College of Medicine, Houston, TX USA

**Keywords:** Preventive screening, Primary care, Workflow, Teamwork, Adaptation, Multi-team systems, Public health emergencies

## Abstract

**Background:**

Screening lies at the heart of preventive care. However, COVID-19 dramatically disrupted routine screening efforts, resulting in excess mortality not directly attributable to COVID-19. Screening rates during COVID varied markedly by facility and clinical condition, suggesting susceptibilities in screening and referral process workflow. To better understand these susceptibilities and identify new practices to mitigate interrupted care, we propose a qualitative study comparing facilities that exhibited high, low, and highly variable performance (respectively) in screening rates before and during the pandemic. We will be guided by Weaver et al.’s multi-team systems (MTS) model of coordination, using cancer and mental health screening rates as exemplars.

**Method:**

Qualitative analysis of interviews and focus groups with primary care personnel, leadership, and patients at 10 VA medical centers. We will select sites based on rurality, COVID-19 caseload at the beginning of the pandemic, and performance on five outpatient clinical performance indicators of cancer and mental health screening. Sites will be categorized into one of five screening performance groups: *high performers*, *low performers*, *improvers*, *plummeters*, and *highly variable*.

We will create process maps for each performance measure to create a workflow baseline and then interview primary care leadership to update the map at each site. We will clinician conduct focus groups to elicit themes regarding clinician coordination patterns (e.g., handoffs), strategies, and barriers/facilitators to screening during COVID. We will also conduct patient interviews to examine their screening experience during this period, for context. All interviews and focus groups will be audio-recorded, transcribed, and enhanced by field notes. We will analyze clinician transcripts and field notes using iterative, rapid analysis. Patient interviews will be analyzed using inductive/deductive content analysis.

**Discussion:**

Our study represents a unique opportunity to inform the multi-team systems literature by identifying specific forms of information exchange, collective problem solving, and decision-making associated with higher and improved clinical performance. Specifically, our study aims to detect the specific points in the screening and referral process most susceptible to disruption and coordination processes that, if changed, will yield the highest value. Findings apply to future pandemics or any event with the potential to disrupt care.

**Supplementary Information:**

The online version contains supplementary material available at 10.1186/s43058-023-00507-2.

Contributions to the literature

- Screening saves lives and conserves resources; our study aims to detect the specific points in the screening and referral workflow most susceptible to disruption and identify specific coordination mechanisms that function as high-value implementation strategies for maintaining prevention services during public health emergencies.
- Findings will provide a nuanced understanding of successful implementation strategies that help facilities adapt how they conduct preventive screening during times of public emergencies and what implementation barriers prevent facilities from adapting.
- Our study expands the multi-team systems literature by identifying specific forms of information exchange, collective problem solving, and decision-making associated with improved implementation success.

## Background

### Changes in preventive screening rates during the COVID-19 pandemic

Preventive screening, where asymptomatic patients are tested to diagnose a disease and (when needed) referred for early treatment, lies at the heart of preventive and high-quality primary care. However, primary care teams during public health emergencies like the COVID-19 pandemic faced sudden, unprecedented changes in staff configuration and workflow to meet pandemic demands, thereby dramatically affecting preventive screening efforts in the US and elsewhere. For example, colorectal and breast cancer screening, which require physical patient contact, saw drastic decreases in screening volume in 2020 [1–3]. Screening for more telehealth-friendly conditions, such as depression and PTSD, however, unfolded quite differently during the pandemic; the very infection control measures required to arrest the transmission of COVID-19 (social isolation, physical distancing) exacerbated mental health concerns and magnified the volume of referrals needed, usually detected through screening [4, 5]. Increased screening volume may have created disruptions in successful referrals to mental health services. Thus, cancer and mental health screening serve as two contrasting, yet illustrative examples of susceptibilities in the screening and referral workflow.

In the Veterans Health Administration (VHA), national rates of colon, breast, and cervical cancer screening as well as depression and PTSD screening remained relatively consistent between the beginning of the pandemic and the present. Individual VHA facilities, however, told a different story. Facility-level analyses of these measures indicated considerable variation across sites pre- and during-pandemic: (1) *high performers* who maintained high levels of screening, (2) *plummeters* that could not maintain high performance post-pandemic, (3) *improvers* that surprisingly increased screening post-pandemic, (4) *highly variable* facilities that exhibited marked within-facility variability in performance over time, and (5) *low performers* that did not change [6]. Traditional quality of care and quality improvement models, developed under ordinary conditions, do not sufficiently account for the screening and health outcome patterns observed during the pandemic. What is needed is a nuanced understanding, beyond what can be discerned by the numbers, of what has changed in how facilities currently conduct preventive screening; what worked, and what barriers prevented facilities from adopting successful implementation strategies to adapt.

### Optimizing workflow is key to avoiding screening and referral disruptions in future emergencies

Facilities can enhance their ability to maintain normal levels of screening and referral during the next pandemic by reconsidering their own work processes [7]. Furthermore, changes to workflows and routines could indirectly impact factors outside the providers’ control; for example, a facility offering mental health screenings and visits through telehealth video could allay patient fears of contracting COVID-19 or overcome transportation barriers for patients. Thus, understanding how primary care teams coordinate among themselves and with specialty care to successfully screen and refer patients to treatment when necessary is an ideal workflow feature to examine.

Fundamentally, screening is a coordinative act; even when screening results are negative, successful preventive screening requires, at minimum, coordination between the ordering provider and the testing staff. For example, a work analysis study of clinical performance measures demonstrated that all screening performance measures studied (including screening for three types of cancer, tobacco use, and depression) involved tasks requiring moderate levels of coordination among multiple clinical staff (primary and/or specialty care, depending on the measure) [8]. A different study found predictability and accountability (key conditions for effective coordination) were key to ensuring that screening results led to successful referrals at two geographically dispersed medical centers [9]. With coordination being central to successfully screening and (when needed) referring patients for treatment, it becomes critical to understand how coordination changed during the COVID-19 pandemic, what implementation strategies were successful, and how these changes led to the observed variability in screening rates among VAMCs.

### Study objectives

Using cancer and mental health screening as exemplars and guided by Weaver et al.’s model of coordination in chronic and complex disease management, our study aims to:

- *Objective 1*. Compare how PACTs from facilities of varying screening performance patterns (high, low, improving, plummeting, variable) during the COVID-19 pandemic coordinated:
  - As a team to conduct screening services
  - With specialty care teams at their facility to conduct screening services
- *Objective 2*. Compare team, facility, and system-based barriers, facilitators, and implementation strategies for continuing screening services during the COVID-19 pandemic among PACTs from VHA facilities of varying screening performance patterns (high, low, improving, plummeting, variable) during that period.

We expect that screening performance will be mostly driven by plans, roles, and routines that lead to improved within-team predictability, accountability, and common understanding, and by boundary spanning, collaborative sensemaking, and entrainment behaviors that facilitate between-team coordination.

## Methods

### Conceptual model: understanding coordination among teams in health care

Our study is guided by Weaver et al.’s model of coordination in chronic and complex disease management (Table 1) [10]. This model blends Okhuysen and Bechky’s model of coordination [11] and the Agency for Healthcare Research and Quality’s (AHRQ) coordination processes framework [12] through a multi-team systems (MTS) lens to explain the inputs, mediators, processes, and outcomes of effective coordination within and between healthcare teams: given that screening involves communicating and sequencing services between primary and specialty care, the Weaver model’s multi-team systems lens provides unique perspective not available in other models of coordination or implementation.

**Table 1:** Weaver et al. framework for care coordination in chronic and complex disease management

	*Context and Setting (moderators or inputs)*	*Coordination mechanisms (Inputs)*	*Emergent integrating conditions (mediators)*	*Coordinating actions (proximal*,* behavioral processes)*	*Outcomes (proximal and distal outcomes)*
Within teams	• Team composition • Experience and history • Power distribution • Resources	• Plans, rules, tools (e.g., standardized care protocols) • Objects, artifacts, information systems, representations	• Accountability • Predictability • Common understanding • Trust	• Situation monitoring • Communication (information sharing and collective sensemaking) • Back-up behavior	• Proximal health outcomes • Proximal care costs • Satisfaction
Between teams	• Multiteam system composition • Linkages between teams • Alignment of organizational cultures/climates • Governance and payment structure			• **Boundary spanning** • **Collaborative sensemaking:** *◦ Information exchange* *◦ Collective problem solving and decision-making* • **Entrainment**: *◦ Negotiation* *◦ Mutual adjustment*	• Distal health outcomes for individual patients (e.g., mortality) • Public health outcomes • Lifetime care costs and value • Satisfaction • Timeliness of care

Okhuysen and Bechky’s context-free model explains the mechanisms and integrating conditions required to coordinate effectively. According to this framework, five basic mechanisms underlie effective coordination: (1) plans and rules (explicit definitions of objectives, responsibilities, and resource allocations; e.g., who is allowed to place an FOBT order?); (2) objects and representations (any device used to create a common referent and create shared meaning; e.g., consult templates); (3) team member roles; (4) routines; and (5) physical proximity among team members. These mechanisms enable teams to achieve three results: (1) accountability (clarity over who is responsible for what), (2) predictability (knowing what tasks are involved and when they happen), and (3) common understanding (providing a shared perspective on the whole process and how individuals’ work fits within that whole). The successful integration of these three conditions allows people to coordinate (collectively accomplish their interdependent tasks). In contrast, AHRQ’s framework identifies 9 activities important for *healthcare* coordination, including assessing needs and goals, creating care plans, communicating, establishing accountability and responsibility for care tasks, facilitating transitions, monitoring and adapting, supporting self-management, linking to community resources, and aligning resources with patient and population needs.

Blending these two models and building on studies of multi-team systems in high-stakes environments, the Weaver model presents 11 teamwork processes important for healthcare delivery, highlighting three key processes that make effective coordination *between* teams (or in our case, between primary and specialty care) possible: (1) boundary spanning (facilitating information flow and managing relationships between groups), (2) collaborative sensemaking (assigning shared meaning to information), and (3) entrainment (mutually adjusting the pace or sequence of tasks based on updates or feedback from other teams).

Its focus on between-team processes makes the Weaver model singularly suited for our study. In examining coordination patterns, mitigating strategies, and barriers and facilitators, we will investigate how teams have altered boundary spanning, collaborative sensemaking, and entrainment to foster predictability, accountability, and common understanding, and thus, trust between primary and specialty care teams.

### Design

Our study consists of qualitative, primary analysis of interviews and focus groups with facility leadership, primary care personnel, and primary care patients at up to 10 VA medical centers (VAMCs), using a double-blind, retrospective design with purposive sampling.

### Defining coordination and the boundaries of screening

In this study, we define coordination as synchronizing and sequencing screening and referral tasks among members of primary care teams and between primary and specialty care [10]. We also define screening broadly to include all activities involved in identifying patient eligibility for screening, completing the screening test or procedure, reporting results, and following up with patients, up to and including referring patients for additional work if the test result is positive. We include results and follow-up because screening is useful only if the clinician reports and acts upon screening results [8].

### Site selection

We will select sites using a purposive stratified approach based on site rurality, sites’ scores on a profile of five outpatient clinical performance, and outpatient COVID-19 positivity rate (OCPR) at the beginning of the pandemic. Definitions and inclusion criteria for each are described below. To select the sites, we will first stratify all VAMCs into rural or urban sites, as rural sites often have markedly different workflows from urban sites and are likely to be naturally underrepresented (85% of VAMCs are located in urban settings) [13]. Within each stratum, we will identify the VAMCs that meet criteria for each of five performance profiles. Applying these two criteria will yield 10 sites. We will examine the resulting sites at this point for their OCPR: ideally, each performance profile should have one site with a low OCPR and one with a high OCPR. Should a given performance category not have one site of each level of OCPR, we will select the next available site from the stratified list until this last criterion is reached—for some performance profiles the site with the low OCPR will be the urban site, for others the rural. Although this strategy will not yield a fully factorial design (2 × 5 × 2), it will still produce rich variation, capturing a wide range of experiences and strategies within time and budget limits.

#### Rurality

We will obtain rurality designation for each site from the VA Site Tracking (VAST) system, who defines rurality according to the rural-urban communing areas (RUCA) system. Facilities in census tracts with at least 30% of their population residing in an urbanized area as defined by the Bureau of the Census (RUCA codes 1.0 or 1.1) are considered urban (*n* = 145); all others are rural (*n* = 26).

#### Screening performance

Screening performance metrics will encompass two preventive clinical areas: cancer screening and mental health screening (see Table 2). Performance measure scores will be extracted from the External Peer Review Program (EPRP) report (available through VSSC), VA’s longest standing, most stable set of clinical performance measures, and part of the Strategic Analytics for Improvement and Learning (SAIL) system used by all VA facilities to summarize hospital system performance. These two clinical areas were selected specifically due to the levels of coordination involved [8, 14] and because they represent two conditions with contrasting yet detrimental effects from the pandemic on demand for screening. The screening performance observation period for site selection purposes will be October 2019–September 2021 (8 quarters). Quarter 3 (Q3) of fiscal year (FY) 2020 (March–May 2020) will be considered the start of the pandemic.

**Table 2:** Clinical performance measures for site selection

**Measure mnemonic**	**Short description**
*Cancer screening*	
• P61h	Colorectal cancer screening: positive fecal occult blood tests (FOBT) that lead follow-up colonoscopy
• P41h	Cervical cancer screening
• P32h	Breast cancer screening
*Mental health screening*	
• Mdd40	Depression screening
• Hc41	Post-traumatic stress disorder (PTSD) screening

Site selection using purposive sampling commonly involves selecting high- and low-performing sites, as it maximizes the researcher’s ability to observe differences among the strata in the construct of interest. In this case, however, a seminal event exists (the pandemic) that materially changes the sites’ reality; this change is, in fact, the phenomenon of interest. Consequently, in addition to high and low performing sites, we will also select sites whose performance before and during the pandemic has significantly improved, significantly plummeted, and exhibited high variability during the pandemic. Consistent with previous research [15], we will select two facilities (one rural, one urban) of each of these five types:

1. *High-performing facilities* are those whose screening scores during the observation period are consistently at the 84th percentile *(%ile)* or higher. In prior research, no single *site* scored above the 84th *%ile* on all measures examined [15]. Consequently, we will sort facilities by the number of EPRP measures meeting this criterion and target the *two* facilities with the greatest number of measures meeting criterion.
2. *Low-performing facilities* are those whose screening scores during the observation period are consistently at the 16th *%ile* or lower. In prior research [15], no single facility exhibited scores below the 16th *%ile* on all the measures examined. We will sort facilities by the number of EPRP measures meeting this criterion and target the *two* facilities with the greatest number of measures meeting this criterion.
3. *Improving facilities* are those whose screening scores during Q1 of the observation period are, on average, at the 16th *%ile* or lower yet exhibited average improvements of 5 percentage points or greater by the end of the observation period (*Q8*) and no significant decreases along the way. A change of five percentage points is considered clinically or operationally significant by VA’s Office of Performance Measurement (Francis, J, personal communication, November 1, 2016). We will select the *two* facilities with the greatest improvements in performance, as exhibited by the slope of the line between Q1 and *Q8*. Should no facilities meet this criterion, we will select facilities with the greatest improvement between Q1 and *Q8* (i.e., the steepest slope), regardless of their percentile in Q1.
4. *Plummeting facilities* are those whose screening scores during Q1 of the observation period are, on average, at the 84th *%ile* or higher but (unlike high-performing facilities) exhibited average decreases *≥* 5 percentage points by the end of the observation period (*Q8*) and no significant increases along the way. We will select the *two* facilities with the greatest declines in performance, as exhibited by the slope of the line between Q1 and *Q8.* Should no facilities meet this criterion, we will select facilities with the greatest decline between Q1 and *Q8* (i.e., the steepest slope), regardless of their *%ile* in Q1.
5. *Highly variable facilities* are *those* facilities whose screening scores exhibit the highest standard deviations across measures and quarters *combined* during the observation period. As research [16] indicates that lower performing facilities tend to exhibit more variability than higher performing sites, we will only include highly variable sites that do not already qualify for one of the other arms.

#### Outpatient COVID positivity rate (OCPR)

The percent of COVID tests resulting as positive at a facility is an important factor that at any time could significantly and detrimentally impact screening rates, and must thus be considered in selecting sites. However, COVID-19 did not spread uniformly over time across the USA, and geographic spread patterns continued to change throughout the life of the pandemic. Thus, we will consider the OCPR between April 1 and April 15 (one full reporting period after the declared COVID-19 a global pandemic) as a site selection criterion. Our rationale is that sites experiencing high rates of COVID early in the pandemic would have had less time to prepare and to learn from other sites and would have likely adapted very differently than sites with more time to prepare. Thus, each performance category will include one site, which could be rural or urban, with a high OCPR and one with a low OCPR (high: > 10%; low: < 5%).

### Participants

#### Clinicians

We will interview the Associate Chief of Staff for Primary Care (ACOS-PC) or their designee at each site to act as a key informant. We will recruit up to 20 employees at each facility to participate in up to 2 focus groups per site (with 6–8 people per group) and individual interviews as needed with up to 5 team members. We will target patient-aligned care team (PACT) members (provider, care manager, care associate, clerk) and relevant specialty care personnel, based on scheduling availability. We will make every attempt to ensure role diversity in each focus group. To ensure data quality, we will target full-time personnel who have worked in their current position for the entirety of the observation period.

#### Veterans

We will recruit up to eight veterans from each facility, to be interviewed in a focus group, selected from the patient panels of the PACTs interviewed at each site. To identify eligible veterans we will generate, for each site, a list of patients who have been screened for one or more of the conditions of interest in the study. Data for this purpose will be drawn from VA’s Corporate Data Warehouse. Preference will be given to patients who have been screened for multiple of the conditions in Table 2. We will also target screening-eligible patients who were not screened during the observation period.

### Recruitment strategy

#### Clinicians

We will recruit personnel using a snowball sampling strategy: we will request recommendations from the ACOS-PC for eligible clinician participants and subsequently request recommendations from participants along the way as needed. Should this strategy not yield sufficient participants, we will supplement it with searches from the primary care Team Assignments Report, available through VSSC. We will confirm eligibility during informed consent and scheduling.

Prospective participants will receive an email inviting them to enroll and requesting a preferred contact email, phone number, and a time where a study coordinator can conduct informed consent procedures. Invitees who have not responded within ten calendar days will receive a follow-up reminder. Research team members will email prospective participants the study information form in advance and will contact them to confirm eligibility, conduct consent procedures, answer questions, and schedule the focus group/interview. One week before the interview, participants will receive an interview preparation guide to help them recall facts and processes that may be temporally distant from the focus group or interview date.

#### Veterans

Eligible veterans will be identified as described in the “Participants” section. Eligible Veterans will be contacted through mail first with a telephone follow-up 10 days following the letter. The introduction letter will include an opt-out plan (e.g., a return envelope and card, an email address, and phone number to respond to). We will over sample (up to 10 Veterans) to account for attrition. Veterans will also receive an interview preparation guide similar to the clinician one, tailored for their focus groups.

### Research team and participant blinding

Interviewers, coders, and participants will be blinded as to what type of facility they are interviewing, coding, or participating in to minimize bias during data collection and analysis [15].

### Data collection

We will use a qualitative approach with multiple methods to explore perceptions and experiences between sites and identify barriers and facilitators to change [17]. Using focus groups, process mapping, and interviews affords the research team flexibility in following and integrating emergent themes from the data. Speaking to both clinicians and veterans will allow a layered understanding of screening processes and benefit from multiple perspectives to explore how clinic contexts shape coordination and care. At each site, we will begin with individual ACOS-PC interviews, followed by clinician and veteran focus groups, respectively. Please see Supplemental File 1 for copies and examples of all our data collection materials, including preliminary focus group guides, sample process maps, and participant pre-interview/focus group preparation materials.

#### Process maps

We will use process maps of each performance measure generated in prior research [8] as a baseline for comparison to the COVID-adapted process being employed now at each site. As the maps are now several years old, we will conduct initial interviews with the ACOS-PC at each site to confirm the maps accurately reflected the workflows used at the facilities before the pandemic; process maps will be amended as needed based on the ACOS-PC input. We will also ask the ACOS-PC to indicate any changes in the process map since the pandemic began and update the maps as needed. The updated (since March 2020) maps will be used as starting points for the clinician focus groups.

#### Focus groups

##### Veterans

At each site, we will conduct one 60-min virtual focus group with Veterans to explore their experiences of screening services during the pandemic, transitions of care from PCP to specialty clinics, and perceptions of care management at the sites during the pandemic. We will use a VA-approved virtual communication platform such as Microsoft Teams or Webex. We will invite Veterans to reach out if they would like to discuss any issue addressed in the focus group further one-on-one concerning discussion of mental health screening in a group setting. Rapid analysis will allow our team to categorize emergent themes along with the participants and adapt as necessary. Findings will be incorporated into our in-depth clinician focus groups to ensure we are exploring Veteran-identified concerns.

##### Clinicians

We will conduct up to two virtual, 60-min clinician focus groups at each site. We will use focus groups to elicit from PACTs the ways in which screening has changed during COVID. Focus groups are most appropriate due to the coordinative nature of screening. Individual interviews would leave it to up to the research team to infer any process gaps; in contrast, focus groups allow for real-time clarification of such gaps, as well as observation of group interactions, range of beliefs, and areas of disagreement among clinicians. At each site, we will discuss focus group makeup with the ACOS-PC (or designee) to determine whether personnel from primary and specialty care can form part of the same focus group or whether separate focus groups should be conducted; this is to ensure focus group dynamics conducive to healthy discussion.

We will use a VA-approved virtual communication platform such as Microsoft Teams or Webex for the focus groups, which will be facilitated by an experienced qualitative methodologist. A research associate will take detailed notes and assist with identifying themes. The focus group will be semi-structured, based on the Weaver et al. (2018) model [10] and our research questions: (1) comparing coordination patterns (a) within primary care teams and (b) between primary and specialty care teams; (2) strategies, barriers, and facilitators to maintaining continued screening services.

#### Interviews

We expect that it will not be possible to explore certain issues in depth in a focus group setting due to the topic, or power dynamics around roles. Therefore, we will conduct follow-up semi-structured interviews when needed, to further explore emergent clinician and Veteran focus group themes and clarify and identify nuance within themes. We will conduct interviews at a site if (a) a participant requests a follow-up interview or (b) a given detail from the focus group requires clarification that could be obtained from a participant. Should a follow-up interview be needed, participants will be contacted in a manner similar to the original recruitment process described earlier. The number of interviews will depend on circumstances at each site; we will plan capacity for up to 5 30-min interviews.

#### Interviewer/facilitator training

To ensure consistency of delivery across interviews and focus groups, the PI will train the experienced team of interviewers on interview/focus group techniques specific to the project, with particular focus on eliciting the coordination constructs of interest. Training will follow the Information, Demonstration, Practice (IDP) framework [18] of training delivery: a didactic training session (information), trainee observation of mock interviews/focus groups, (demonstration), and two mock interviews (practice).

### Data analysis

#### Process maps

As discussed earlier, each site’s updated maps will be used as a jumping off point for focus groups. The maps will also be compared across facilities and across measures to identify key components of the screening workflow that may require attention. To accomplish these comparisons, the steps depicted in each process map for each measure at each site will be entered in a spreadsheet. Each row (record) will constitute one step (e.g., place order for screening test in EHR); each column will constitute an attribute of that step (e.g., measure, facility, performance category of the facility, who performs the step, sequential order). Once transformed from their normal visual format to a spreadsheet, the data can be analyzed like any other qualitative or quantitative dataset. We will start by identifying any key steps that are common to all measures (signifying a critical piece of the workflow, and that facilities should make sure is done efficiently and effectively). We will then conduct cross-site comparisons of each measure, which will include the following: (a) extent of process variability across sites for a given measure, assessed descriptively through average number of steps for a measure and its standard deviation; (b) extent of process variability for a given measure across performance category (e.g., consistent with prior research on clinical practice guideline implementation [19], it is possible that the adapted process used by all high performers for a measure is approximately similar, whereas low performing sites exhibit high process variability); (c) were there key steps in a measure that changed (i.e., either altered or deleted entirely) or remained unchanged across a majority of sites?

#### Focus groups

All focus groups will be audio-recorded, transcribed verbatim, and enhanced by field notes/observations. We will analyze clinician focus group transcripts and field notes using iterative, rapid analysis [20]. The rapid qualitative analysis approach is designed to deliver timely findings with methodological rigor. This methodology is particularly appropriate to allow our multidisciplinary team to quickly identify areas in need of further exploration with individual clinicians [21] and disseminate findings quickly [22]. A summary template based on the Weaver et al. model will be created and tested by the team with one focus group transcript. Focus group transcripts will be summarized using the template based on the key areas of interest, emergent themes (I.e., categories identified/created with participants), and key observations. Team members will familiarize themselves with the transcript and complete a summary template independently (estimated turnaround time: 2 h per transcript). The team will meet to discuss the summary and to review similarities and differences between researchers’ summaries. Team insights will be consolidated into a single summary. We will repeat this process as a group with the focus group transcripts until consistency has been established. The transcripts will be then divided between the team to complete the summaries. Following completion of all focus groups, data will be visualized in matrix form. The team will meet to discuss the findings and identify areas of additional focus for the semi-structured interviews as needed.

#### Interviews

Interviews will be audio-recorded and transcribed verbatim. As semi-structured interviews are intended to be exploratory, for clarification where needed, transcripts will be analyzed using content analysis. We will use Atlas.ti for data management and analysis. The study methodologists will familiarize themselves with all data as it is received (transcribed) and create an initial codebook, using inductive and deductive coding. Deductive coding will be based on the Weaver model. A 10% sample of the interview transcripts will be independently coded, and reviewers will meet to refine the codebook before applying it to the rest of the interviews. Transcripts will then be independently coded, and any newly emergent codes will be added as necessary. Final coding will be merged and reviewed for disagreements. All discrepant coding will be resolved through team discussion and consensus.

#### Coder training

Before the coding process begins, our senior methodologist will conduct a training session (consistent with the IDP framework) with the coders and co-investigators to familiarize them with the Atlas.ti software and the initial coding taxonomy. The session will consist of two modules:

- A didactic module, where trainees will receive detailed information about the specific a priori codes to be searched for in the texts (e.g., definitions, examples, negative cases), guidelines for identifying new themes and codes, and a demonstration of the Atlas.ti software features and its project-specific use.
- A practice module, where coder teams will use the mock interviews from their interviewer training practice module to practice coding and calibrate the coders to the taxonomy of utility perceptions, strategies, and data-sharing practices. In addition, coders will independently code two transcripts and the team will convene to discuss coding decisions, to further calibrate the coders on live data.

### Expected findings

Based on our prior work, we expect to see considerable variation across sites in the content of the process maps for each given measure. Furthermore, we expect this variation is likely to be a function of interfacility differences in the contextual elements for the Weaver model—plans, roles, and routines that lead to improved within-team predictability, accountability, and common understanding, and by boundary spanning, collaborative sensemaking, and entrainment behaviors that facilitate between-team coordination. For example, it may be possible that all low performing facilities have difficulty making the handoff to schedule a screening test due to inadequate information exchange systems with specialty care. Similarly, we would expect that high performing and improver sites would exhibit evidence of better predictability, accountability, and common understanding.

### Anticipated limitations

#### Timeline feasibility

Hysong and colleagues [23] estimate an average of 24 calendar days from initial contact to scheduling of clinician participants for individual interviews; this estimate is likely longer for focus groups due to the need to coordinate multiple schedules for a single meeting. If data were to be collected strictly sequentially, 10 focus groups would take nearly a year to collect. To make our study feasible within the proposed timeline, our team contains 6 people (paired up into 3 2-person teams) equipped to conduct a focus group at any given time. We are thus able to conduct up to three focus groups (and their analyses) in parallel at any given time. Furthermore, we have designed our recruitment strategy to prioritize performance category (e.g., improver, plummeter) over site or role. This allows us to check for thematic saturation concurrently, which may enable results with fewer than 10 sites.

#### Recall bias

As our proposed study is necessarily retrospective in design, there is risk that participants may inaccurately recall the events of interest to our study and provide biased responses to our questions. The focus group design, however, provides some protection against this bias, as other members of the focus group can correct or add detail to a response from a participant, in essence forming a transactive memory system [24] and in turn reducing inaccuracies. The semi-structured approach, where questions are broad yet still directed at eliciting information about specific constructs, can also help mitigate this concern. Finally, participants will receive an interview preparation guide in advance of their interview to help them better recall facts and processes that may be temporally distant from the focus group or interview date.

### Study status

We have received IRB approval, selected study sites, and are currently in the process of recruiting ACOS-PCs for initial informant interviews. Our original site selection strategy did not yield sufficient candidate sites. Supplemental File 2 describes the adaptations made to our site selection criteria to ensure sufficiency of sites.

## Discussion

Screening and early prevention saves veteran lives and conserves resources during an already resource-exhausting pandemic. Disruptions in screening and referral could happen at any point in the process, from noticing the need for screening, to scheduling the screening appointments, to conducting the screening, to referring patients for care if needed. Current performance measures can tell us whether a problem exists but cannot tell us where the disruption lies, what contextual factors are causing the disruption, nor what barriers must be removed, changes made, or new interventions implemented to ensure smooth operations and reliable care. Our study aims to detect the specific points in the screening and referral process most susceptible to disruption. Our study will also identify specific coordination processes and mechanisms that, if changed, will yield the highest value. Findings from the study are applicable not only to future pandemics but to any event with the potential to disrupt care, such as implementation of new EHRs (Cerner), staff turnover, or natural disasters (e.g., the next hurricane Maria or tropical storm Harvey).

### Implications for implementation science

In their latest primary care consensus report [25], NASEM identifies preventive screening as a core function of primary care, including screening for conditions normally treated in specialty care (e.g., cancer, mental health disorders). They also highlight the team-based nature of screening, noting the multiple members of the primary care team responsible for some form of screening (e.g., physicians, nurses, social workers). VA has delivered team-based primary care for over a decade. As the largest integrated healthcare system in the country and as part of a federal agency, VA is uniquely positioned to create a high-reliability system of preventive care that can maintain continuity during any emergency or seminal event. Much like parts of the national power grid can draw from other areas during times of great need, primary care workflow processes can be redesigned flexibly to maintain continuity of services during times of crisis. Our study can inform the design of such a workflow.

In addition, our research represents a unique opportunity to inform the MTS literature by identifying specific forms of information exchange, collective problem solving, and decision-making associated with higher and improved clinical performance. For example, when a provider receives the results of a screening test from a specialty service and is deciding on next steps, does it matter who reported the results? MTS theory predicts that if the MTS is functioning properly, the provider could credibly receive test results from anyone on the specialty team; this makes it far easier for the workflow to be sustained when routines are disrupted. As MTS research is still nascent in healthcare, our research fills an important scientific gap and informs facility leaders on how to adapt their workflows to maintain continuity and quality during public health and other emergencies.

### Supplementary Information

**Additional file 1: Supplemental File 1.** Data Collection Materials.

**Additional file 2: Supplemental File 2.** Adaptations to Site Selection Strategy

## Data Availability

Not applicable—no data are contained in this study protocol paper.
