# Supplementary material for: Study protocol: maintaining preventive care during public health emergencies through effective coordination
Source: Implement Sci Commun. 2023 Nov 27;4:150. doi: 10.1186/s43058-023-00507-2 (PMC10680205; doi:10.1186/s43058-023-00507-2)
Supplement: Supplementary file 1 — Additional file 1: Supplemental File 1. Data Collection Materials. [file 43058_2023_507_MOESM1_ESM.docx]

# Supplemental File 1: Data Collection Materials

**Note:** The following data collection guides are intended to be used as semi-structured interview and focus guides. The questions in the guides are intended to be relatively broad, eliciting more discussion from participants than more structured interview and focus group guides. Further, the interviewers and focus group facilitators may ask additional follow-up questions based on the participants’ responses to the questions in the guide. In addition, though the guides do not explicitly reference the Weaver (2018) model or its constructs, the questions were designed to elicit responses related to the model and the Weaver model will be the primary coding framework for the analysis of the resulting transcripts.

**Contents:**

1. Pre-Interview Preparation Guide for Participants
2. ACOS-PC interview guide
3. Clinician focus group guide
4. Veteran focus group guide
5. Example process map

**Note:** The materials below are presented exactly as they were presented in our proposal for research funding.

All materials below are preliminary and will be refined as needed as we begin data collection and have a better grasp of local conditions at the participating sites. The questions are deliberately broad so as to elicit natural responses and not overly or artificially lead participants toward the specific coordination constructs of interest.

## Pre-Interview Preparation Guide for Participants

**Note:** This preparation guide will be sent to participants when the research team schedules their interview date/time, and then again as a reminder a few days before their scheduled interview (e.g., a week in advance). The idea is to help them understand what will be asked of them and give them the opportunity to search their records in advance if needed, to aid with recall (particularly with some of events early in the pandemic).

Thank you for agreeing to speak with us about the way your PACT team has been affected by the COVID-19 Pandemic. We want to understand the effect of the Pandemic on coordination within your team and with other PACT teams. We are especially interested in the way that changes in team coordination affected cancer and mental health screening. We have created this guide to help you prepare for your interview.

To prepare to talk about your experience, we would like for you to think about the way you and members of your team have adapted to challenges presented throughout the Pandemic. Please use the questions and prompts below to help you remember some of the changes that have taken place.


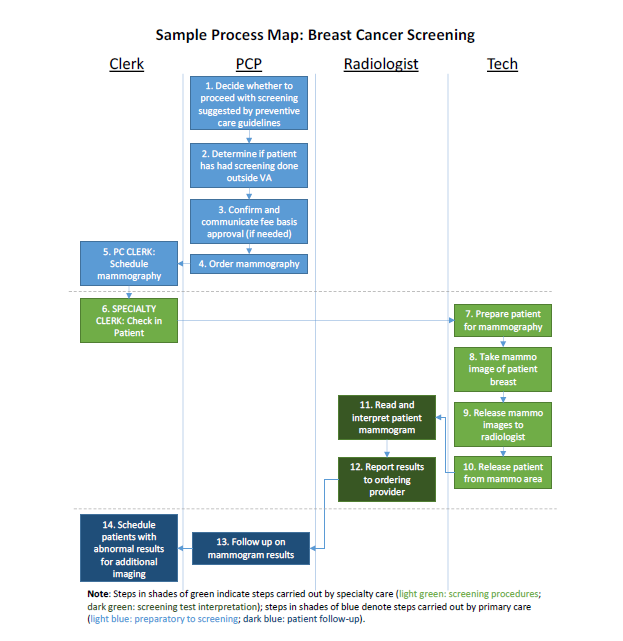


Figure 1. Sample Process Map for Breast Cancer Screening

**Part 1: Think about your role and the work you do in the screening process.**

During the focus group, we will ask you questions to help create a process map like the one shown here. The purpose of this map is to visualize who on the team takes action and when that action takes place. Think about where you fit into this picture.

- What does it look like when your team works to get a patient screened for cancer or mental health conditions?
- What does each team member do?
- How have your team’s screening processes changed since the start of the Pandemic? For the purposes of our discussion, we are signifying April 1^st^, 2020, as the beginning of the Pandemic.

Can you describe what the Pandemic timeline looked like at your facility? For example, when was the beginning, peak, and ultimately fading out of the Pandemic?

**Part 2: Think about how your team works together**

As you recall your experience, also consider these questions:

- How do members of your team communicate with others, such as specialists, during the screening process?
- How has coordination among teams changed throughout the Pandemic?

## Preliminary ACOS-PC Interview Guide

*Thank you again for taking the time to speak with us today. The purpose of this interview is to help us get a better sense of how team coordination within PACT teams and between PACT teams and others at the hospital may have been impacted by the COVID 19 Pandemic. We are especially interested in the way team coordination has affected cancer and mental health screening services.*

*Please note that your participation in this interview is voluntary. If there is a question that you do not wish to answer, please say so and we will move on to the next one. If at any time you decide you no longer want to continue with the interview, we will stop.*

*With your permission, we will be recording this interview so we can make sure our notes are accurate. Because you are one of only a few individuals taking part in this study, there is a risk of a loss of confidentiality, though measures have been taken to minimize this risk. Also, interviews requesting candid responses about oneself may be uncomfortable or stressful, however any risk of this type of discomfort is minimal as you will be answering questions about clinical practices at your facility rather than about personal information.*

*Before we begin, do you have any questions for me?*

If YES, please answer questions. If you are unable to answer the question(s) accurately, please inform the participant: *I will ask the PI (Dr. Hysong) or the study coordinator (Richard SoRelle) about your question(s), and I’ll get back with you with an answer.*

*Your verbal consent to participate in this interview means that you have received this information about the study and that you agree to volunteer for this research study. Please state your name and today’s date to confirm that you agree to participate in this research.*

**Questions**

1. We are interested in the way PACT teams at your facility have changed their process of routine screening for cancers and mental health conditions during COVID-19. For the purpose of our conversation, we are signifying April 1^st^, 2020, as the beginning of the Pandemic. I am going to show you a process map, a visual representation of the individual tasks involved in completing (select different process maps for cancer screenings or mental health screenings). This map was created using research done before the Pandemic.

*Probes*

Think back to how you screened patients for <insert condition here> before the pandemic. Does this map represent the process reasonably accurately? Please point out any differences in the process.

- 1. Now, think about how patients were screened during the Pandemic. What changes would need to be made to the map to represent the process accurately?
  2. Are changes in the process the result of changes to facility or national policies?
  3. Please locate potential challenges (bottlenecks, etc.) in the process on this map (if any)?
  4. Describe any special practices or workarounds your facility uses to address challenges to screening processes.
  5. Finally, think about how patients are screened now. What does the process look like currently?

1. Are there any specific challenges that you faced with regard to managing screening in Primary Care at your facility during the Pandemic?
   1. How has your facility dealt with those challenges?
   2. Who or what has helped you solve problems with screening procedures?
   3. Have any aspects of screening changed for the better?
2. How has the practice of Primary Care at your facility changed as a result of COVID 19?

*Probes*

- 1. Has the composition of PACTs changed during the Pandemic?
  2. How has the distribution of responsibilities (*could be alternately explained as “who is responsible for what”)* amongst PACT members changed during the Pandemic?
  3. How did PACT members respond to changes in their roles during the Pandemic?
  4. To what extent has the availability of non-VA screening options impacted screening services during the Pandemic

*Thank you for your answers. As our goal is to better understand the ways in which the COVID-19 Pandemic impacted the coordination of primary care teams, we wanted to ask you:*

1. Which other individuals at your facility should we talk to about screening and team coordination during the Pandemic?
   1. Is there a PACT that coordinated screening particularly well together during the Pandemic?
   2. Is there a PACT that struggled to coordinate screening effectively during the Pandemic?
   3. Who, in particular, within those PACTs should we speak with?
2. Is there anything else that you would like us to know about PACT performance related to cancer and mental health screening within Primary Care during the pandemic?

**Grounded Probes**

*Use the following probes to elicit further explanation from the above questions by including the following phrasing with the “blanks” filled in by verbatim phrases from the participant.*

- What do you mean by___?
- Can you tell me more about___?
- Can you give me an example of___?
- Can you tell me about a time when___?
- Can you tell me who___?
- Can you walk me through___?
- When___?
- Where___?

## Clinician Focus Group Guide

*Thank you again for taking the time to speak with us today. The purpose of this focus group is to help us get a better sense of how team coordination within PACT teams and between PACT teams and others at the hospital may have been impacted by the COVID 19 Pandemic. We are especially interested in the way team coordination has affected cancer and mental health screening services.*

*Please note that your participation in this focus group is voluntary. If there is a question that you do not wish to answer, you are not required to respond. If at any time you decide you no longer want to continue, you may leave the group.*

*With your permission, we will be recording this focus group so we can make sure our notes are accurate. Because you are one of only a few individuals taking part in this study, there is a risk of a loss of confidentiality, though measures have been taken to minimize this risk. Also, focus groups requesting candid responses about oneself may be uncomfortable or stressful; however, any risk of this type of discomfort is minimal because you will be answering questions about clinical practices at your facility rather than about personal information.*

*Before we begin, do you have any questions for me?*

If YES, please answer questions. If you are unable to answer the question(s) accurately, please inform the participant: *I will ask the PI (Dr. Hysong) or the study coordinator (Richard SoRelle) about your question(s), and I’ll get back with you with an answer.*

*Your verbal consent to participate in this interview means that you have received this information about the study and that you agree to volunteer for this research study. Please state your name and today’s date to confirm that you agree to participate in this research.*

**Questions**

1. We are interested in the way PACT teams at your facility have changed their process of routine screening for cancers and mental health conditions during COVID-19. For the purpose of our conversation, we are signifying April 1^st^, 2020, as the beginning of the Pandemic. First and foremost, thank you for taking the time to review the process maps. This is what the process maps look like after receiving everyone’s input. Are there any final changes we may have missed?

*Probes*

- 1. Please point out differences in pre-COVID, early pandemic, and current processes. Again, for the purpose of our conversation, we are signifying April 1^st^, 2020, as the beginning of the Pandemic. *(note to interviewer: Use the various versions of the process maps resulting from the ACOS interviews to help guide the discussion)*

1. With regard to cancer and mental health screening, how do PACTs coordinate with one another and with specialists?

*Probes*

- 1. How have communication practices changed throughout the Pandemic?
  2. What resources have been necessary to maintain communication related to screening between PACT members?
  3. Which changes are most important to you and your team?
  4. How have changes affected your daily work?
  5. What kinds of challenges have these changes presented?
  6. What resources have been necessary to maintain communication between different PACTs or between PACTs and specialists?

1. Are there any other specific challenges that your team faced with regard to your screening process in Primary Care at your facility during the Pandemic?

*Probes*

- 1. How has your facility dealt with those challenges?
  2. Who or what has helped you solve problems with screening procedures?
  3. Have any aspects of screening changed for the better?
  4. Which changes are most important to you and your team?
  5. How have changes affected your daily work?
  6. What kinds of challenges have these changes presented?

1. How has the practice of Primary Care at your facility changed due to COVID 19?

*Probes*

- 1. Has the composition of PACTs changed during the Pandemic?
  2. How has the distribution of responsibilities (*could be alternately explained as “who is responsible for what”)* amongst PACT members changed during the Pandemic?
  3. Tell us about any challenges or benefits your team experienced because of these changes.
  4. Which changes are most important to you and your team?
  5. How have changes affected each of you personally?

**Grounded Probes**

*Use the following probes to elicit further explanation from the above questions by including the following phrasing with the “blanks” filled in by verbatim phrases from the participant.*

- What do you mean by___?
- Can you tell me more about___?
- Can you give me an example of___?
- Can you tell me about a time when___?
- Can you tell me who___?
- Can you walk me through___?
- When___?
- Where___?

## Veteran Focus Group Guide

*Thank you again for taking the time to speak with us today. The purpose of this focus group is to help us get a better sense of how your healthcare may have been impacted by the COVID 19 Pandemic. We are particularly interested in the way cancer and mental health screening services may have changed during that time.*

*Please note that your participation in this focus group is voluntary. If there is a question that you do not wish to answer, please say so and we will move on to the next one. If at any time you decide you no longer want to continue with the focus group, we will stop.*

*With your permission, we will be recording this focus group so we can make sure our notes are accurate. Because you are one of only a few individuals taking part in this study, there is a risk of a loss of confidentiality, though measures have been taken to minimize this risk. Also, focus groups requesting candid responses about oneself may be uncomfortable or stressful, however any risk of this type of discomfort is minimal as mostly you will be answering questions about clinical practices at your facility.*

*Before we begin, do you have any questions for me?*

If YES, please answer questions. If you are unable to answer the question(s) accurately, please inform the participant: *I will ask the PI (Dr. Hysong) or the study coordinator (Richard SoRelle) about your question(s), and I’ll get back to you with an answer.*

*Your verbal consent to participate in this focus group means that you have received this information about the study and that you agree to volunteer for this research study. Please state your name and today’s date to confirm that you agree to participate in this research.*

**Questions**

1. Describe your Primary care experience at VA.
   1. How long have you been using VA Primary Care?
   2. Is it different than care at other places (if it applies to you)?
2. Show of hands: have you been screened for cancer or a mental illness? (*List relevant cancer or mental health screening procedures to prime responses. For mental health screenings:* The VA does require all patients receive mental health screenings. How many of you recall being asked [screening question] or a similarly phrased question?)
   1. Anyone who is willing, please walk us through your experience.
   2. Did you experience any challenges during the screening?
   3. What concerns, if any, did you have during the process?
3. Has anyone been screened for cancer or a mental illness during the Pandemic? For the purposes of our discussion, we are signifying April 1^st^, 2020, as the beginning of the Pandemic.
   1. Anyone who is willing, please walk us through your experience.
   2. Did you experience any challenges during the screening?
   3. What concerns, if any, did you have during the screening process?
4. Has anyone been screened at a non-VA clinic during the Pandemic?
   1. Anyone who is willing, please walk us through your experience.
   2. Did you experience any challenges during the screening?
   3. What concerns, if any, did you have during the process?
5. Please tell us anything else you want me to know about your Primary Care or screening experience at VA.

**Grounded Probes**

*Use the following probes to elicit further explanation from the above questions by including the following phrasing with the “blanks” filled in by verbatim phrases from the participant.*

- What do you mean by___?
- Can you tell me more about___?
- Can you give me an example of___?
- Can you tell me about a time when___?
- Can you tell me who___?
- Can you walk me through___?
- When___?
- Where___?

## Sample Process Map

Below is an example of the process map for Breast Cancer Screening developed from Dr. Hysong’s prior research. Process maps such as these will be used as part of the ACOS-PC and clinician focus groups as described in sections 2E.6 and 2E.7 of the research plan.
