# Supplementary material for: Study protocol: maintaining preventive care during public health emergencies through effective coordination
Source: Implement Sci Commun. 2023 Nov 27;4:150. doi: 10.1186/s43058-023-00507-2 (PMC10680205; doi:10.1186/s43058-023-00507-2)
Supplement: Supplementary file 2 — Additional file 2: Supplemental File 2. Adaptations to Site Selection Strategy [file 43058_2023_507_MOESM2_ESM.docx]

# Supplemental File 2 – Adaptations to Site Selection Strategy

| **Performance Category** | **Obstacle(s) Encountered with Selection Criteria** | **Adaptations Made** |
| --- | --- | --- |
| High-performing facilities | Criteria were too strict – 15 sites met the criteria but only for 1 of 5 performance measures | We relaxed the percentile to 75% and selected the two sites with the greatest number of measures that met this percentile in at least 5 of the 8 quarters. We required a minimum of 3 measures to meet the criteria |
| Low-performing facilities | Criteria were too strict – 3 sites met the criteria but only for 1 of 5 performance measures | We relaxed the percentile to 25% and selected the two sites with the greatest number of measures that met this percentile in at least 5 of the 8 quarters. We required a minimum of 3 measures to meet the criteria |
| Improving facilities | Multifaceted criteria were too strict (especially the criteria of no decreases >5% points between quarters) and resulted in no sites meeting all criteria | We selected sites whose measures that had the greatest average positive linear slope (across the 8 quarters) that also met the other criteria reasonably well (i.e., at least 3 measures started in the 25th percentile or lower/none in the 75th percentile or higher, at least 3 measures exhibited improvement of at least 5% points (relative to Q1) by Q8, and fewer than 10 instances where scores decreased by 5 percentage points or more between quarters). |
| Plummeting facilities | Multifaceted criteria were too strict (especially the criteria of no increases >5% points between quarters) and resulted in no sites meeting all criteria | We selected sites whose measures that had the greatest average negative linear slope (across the 8 quarters) that also met the other criteria reasonably well (i.e., at least 3 measures started in the 75th percentile or higher/none in the 25th percentile or lower, at least 3 measures exhibited a decrease of at least 5% points (relative to Q1) by Q8, and fewer than 10 instances where scores increased by 5 percentage points or more between quarters). |
| Highly variable facilities | Resulted in sites with variable performance between measures but not necessarily variable performance within measures over time | We first calculated the overall variance (measures and quarters combined) for each site and identified those with the largest variance. We selected sites with the greatest overall variance who also had at least higher variance (100) on 1 measure and at least adequate variance (20) on 3 of the 4 other measures. We also considered whether there was just one quarter for a measure greatly contributing to the greater variance and opted for sites with measures with regular/consistent variance where possible. |
| Selection by rural/urban and high/low outpatient covid positivity rate (OCPR) | Given the smaller number of rural site and the smaller number of sites with high OCPRs, performance categories did not always have an acceptable rural site and/or did not always have an acceptable site with high OCPR. | For each performance bucket, we attempted to select one rural and one urban site as well as one site where the outpatient covid positivity rate was high and one site where it was low. In the event that these criteria would have resulted in selection of a sub-optimal site, the criteria were waived (e.g., two urban sites selected for a given performance bucket). Graphs of performance over time for each measure for each of the top sites in the 5 performance buckets were also reviewed for context. |
